# Enhancing Security Operations Center: Wazuh Security Event Response with Retrieval-Augmented-Generation-Driven Copilot

**DOI:** 10.3390/s25030870

**Published:** 2025-01-31

**Authors:** Ismail, Rahmat Kurnia, Farid Widyatama, Ilham Mirwansyah Wibawa, Zilmas Arjuna Brata, Ukasyah, Ghitha Afina Nelistiani, Howon Kim

**Affiliations:** 1 School of Computer Science and Engineering, Pusan National University, Busan 46241, Republic of Korea; ismail@pusan.ac.kr; 2 SmartM2M Co., Ltd., 701, 702, Building A, Centum Skybiz, 97 Centumjungang-ro, Haeundae-gu, Busan 48058, Republic of Korea; rahmat@smartm2m.co.kr (R.K.); farid@smartm2m.co.kr (F.W.); ilham@smartm2m.co.kr (I.M.W.); zilmas@smartm2m.co.kr (Z.A.B.); ukasyah@smartm2m.co.kr (U.); githa@smartm2m.co.kr (G.A.N.)

**Keywords:** security operation center, Wazuh, copilot, incident response management, large language model, retrieval-augmented generation, MITRE ATT&CK, NIST standard

## Abstract

The sophistication of cyberthreats demands more efficient and intelligent tools to support Security Operations Centers (SOCs) in managing and mitigating incidents. To address this, we developed the Security Event Response Copilot (SERC), a system designed to assist analysts in responding to and mitigating security breaches more effectively. SERC integrates two core components: (1) security event data extraction using Retrieval-Augmented Generation (RAG) methods, and (2) LLM-based incident response guidance. This paper specifically utilizes Wazuh, an open-source Security Information and Event Management (SIEM) platform, as the foundation for capturing, analyzing, and correlating security events from endpoints. SERC leverages Wazuh’s capabilities to collect real-time event data and applies a RAG approach to retrieve context-specific insights from three vectorized data collections: incident response knowledge, the MITRE ATT&CK framework, and the NIST Cybersecurity Framework (CSF) 2.0. This integration bridges strategic risk management and tactical intelligence, enabling precise identification of adversarial tactics and techniques while adhering to best practices in cybersecurity. The results demonstrate the potential of combining structured threat intelligence frameworks with AI-driven models, empowered by Wazuh’s robust SIEM capabilities, to address the dynamic challenges faced by SOCs in today’s complex cybersecurity environment.

## 1. Introduction

Over the past decade, the role of Security Operations Centers (SOCs) has grown significantly due to the increasing frequency and sophistication of cyberattacks and the substantial costs associated with data breaches and service disruptions. However, SOCs still face challenges such as a shortage of skilled personnel [1], limited automation [2], and inconsistencies in process integration [3]. Additionally, SOCs must balance rapid technological advancements with continuous staff training and retention requirements [4,5]. The heavy reliance on Security Information and Event Management (SIEM) systems has also introduced issues like high false-positive rates and manual analysis burdens [6], creating operational challenges [7] for traditional SOCs [8].

The complementary adoption of NIST’s strategic breadth and MITRE’s tactical detail allows organizations to build a multi-layered defense that integrates high-level risk management with actionable intelligence [9,10]. The NIST Cybersecurity Framework emphasizes a lifecycle approach to managing cybersecurity risks, spanning essential functions like Identify, Protect, Detect, Respond, and Recover [11]. Whereas, the MITRE ATT&CK framework provides a granular understanding of adversary behaviors, offering a knowledge base for mapping attack methodologies and developing tailored defenses [12].

Furthermore, AI has introduced transformative changes in cybersecurity, enabling more proactive, automated incident responses. AI-driven systems enhance the speed and accuracy of detecting and mitigating incidents by leveraging machine learning and natural language processing for automated threat analysis [13]. Additionally, AI has the potential to orchestrate response processes that improve business continuity and streamline recovery in cyberincidents [14]. These advancements highlight AI’s critical role in addressing traditional incident response’s scalability and accuracy challenges.

Many enterprises are increasingly adopting open-source Security Operations Center (SOC) platforms as cost-effective alternatives to proprietary solutions. This shift is largely driven by budget constraints and the desire for access to a broader range of features. Open-source SOC tools offer robust and feature-rich capabilities, enabling organizations to establish effective security postures without incurring substantial costs [15]. Additionally, the flexibility and customization offered by open-source solutions allow enterprises to tailor their security operations to specific needs, further enhancing their appeal [16].

Despite its cost advantages and flexibility, the open-source SOC ecosystem faces limitations in AI-driven innovation. Wazuh [17], an open-source SIEM platform, has gained recognition for its flexibility, scalability, and powerful threat detection capabilities, making it a viable, cost-effective solution for organizations of various sizes [18]. Wazuh’s compatibility with SOC environments allows seamless integration and enhanced threat monitoring [19]. However, existing implementations of Wazuh in SOCs lack direct integration with advanced AI capabilities, which could significantly improve its detection accuracy and responsiveness.

In addressing these limitations, this paper contributes to the field by (1) enhancing threat data extraction methodologies using RAG, (2) embedding AI within Wazuh for improved incident response by giving mitigation guidance, (3) employing LLMs for precise, context-driven recommendations, and (4) comparing two different models [20,21] to evaluate performance outcomes. These contributions enhance the capabilities of Security Operations Centers (SOCs), advancing modern incident response practice’s adaptability, depth, and effectiveness.

## 2. Background and Related Works

### 2.1. Security Event Definition and Flow

**Figure 1:**
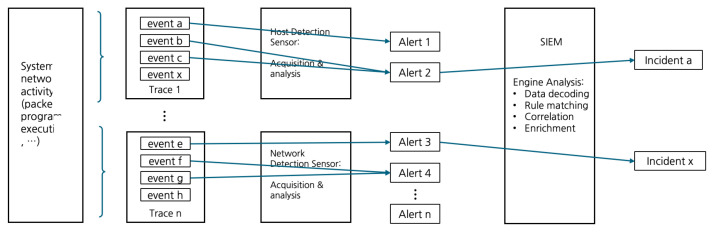
Event security management lifecycle (adapted from [22]).

Event security management is essential for effective cybersecurity operations, providing a structured, iterative process that transforms raw event data into actionable insights for incident response. As illustrated in Figure 1, this lifecycle strengthens an organization’s security posture by systematically detecting, analyzing, and escalating potential security incidents [22].

The process begins with event detection, where tools such as SIEM systems and Intrusion Detection Systems (IDS) monitor logs, network traffic, and user behavior to identify anomalies [23]. Detected events are then systematically logged, creating detailed records that support immediate analysis and future forensic investigations [24].

To manage the large volume of data generated, event filtering is employed to eliminate false positives and benign activities. This ensures that attention is focused on meaningful security signals. Subsequently, events are categorized by severity and type, prioritizing high-risk incidents for response [25]. This stage is crucial for identifying complex, multi-vector threats that isolated data might not reveal. Once a significant risk is identified, incident escalation ensures a timely response, equipping teams with detailed, context-rich information to guide mitigation efforts [23].

### 2.2. Wazuh as a Versatile SIEM Solution

In the realm of Security Information and Event Management (SIEM) solutions, various platforms offer unique features tailored to meet diverse organizational requirements. Table 1 presents a comparative analysis of Wazuh, IBM Security QRadar, Splunk Enterprise Security, Security Onion, and Graylog, focusing on key aspects such as cost, log management capabilities, file integrity monitoring (FIM), integration features, ease of deployment, scalability, and community support.

Among the diverse range of Security Information and Event Management (SIEM) tools available, Wazuh has emerged as a prominent choice due to its adaptability, scalability, and powerful threat detection capabilities. As an open-source platform, Wazuh offers a cost-effective alternative to proprietary solutions, providing organizations with customizable options for managing cybersecurity risks across environments ranging from small businesses to large enterprises [33].

Wazuh stands out by eliminating licensing fees while delivering comprehensive log management capabilities. Its support for diverse data sources ensures robust monitoring across various systems and networks. Additionally, Wazuh’s built-in File Integrity Monitoring (FIM) enables real-time detection of unauthorized changes, a critical feature for maintaining system integrity and compliance. Seamless integration with the Elastic Stack [34] enhances Wazuh’s capabilities for data visualization and analysis, facilitating comprehensive security monitoring and incident response. Although deploying Wazuh requires initial configuration, its flexibility and scalability make it suitable for organizations of different sizes. Furthermore, its active open-source community ensures continuous development and innovation [33].

In contrast, proprietary solutions such as IBM Security QRadar and Splunk-SIEM incur high licensing costs, which can be prohibitive for budget-conscious organizations. While these platforms provide advanced analytics and broad integration capabilities, additional investments may be required to enable features like FIM. Open-source alternatives like Security Onion and Graylog share Wazuh’s cost benefits but face limitations in extensibility and scalability, making them less versatile for diverse organizational needs [35].

Comparative analyses have demonstrated Wazuh’s effectiveness relative to proprietary systems, particularly in areas such as threat detection and operational flexibility. For example, a study comparing IBM QRadar and Wazuh highlighted Wazuh’s ability to meet standard security requirements without the financial burden associated with commercial solutions [36]. User reviews further emphasize the value of Wazuh’s open-source model, citing its customizable rule sets, strong community support, and effective security monitoring [28].

These evaluations affirm Wazuh’s positioning as a competitive SIEM tool that delivers robust cybersecurity functions within a scalable, open-source framework. Its cost-effectiveness, comprehensive features, and adaptability make it a compelling choice for organizations seeking a reliable and efficient security solution.

Recent advancements in artificial intelligence (AI) and machine learning (ML) present significant opportunities to further enhance Wazuh’s capabilities, particularly in incident response and threat intelligence. Research indicates that the application of large language models (LLMs) and AI-driven methodologies can streamline security operations by automating complex analytical processes and delivering real-time, contextually relevant insights. For instance, studies suggest that integrating LLMs within Security Operations Center (SOC) workflows has the potential to improve response accuracy, facilitate rapid decision-making, and mitigate analyst fatigue during incident response [37]. Additionally, other research has explored the potential for LLMs to provide dynamic, context-aware recommendations in incident response scenarios, thereby augmenting Wazuh’s operational efficacy through intelligent, real-time guidance [38].

### 2.3. MITRE ATT&CK Framework

The MITRE ATT&CK (Adversarial Tactics, Techniques, and Common Knowledge) framework is a globally recognized knowledge base that provides a detailed taxonomy of adversarial behaviors observed during cyberattacks. Introduced in 2013 as part of MITRE’s Fort Meade Experiment, ATT&CK categorizes tactics, techniques, and procedures (TTPs) used by adversaries, enabling security professionals to model, analyze, and mitigate cybersecurity threats effectively [10,39].

Structured to capture the entire lifecycle of a cyberattack, the ATT&CK framework organizes adversarial activities into tactics—high-level objectives such as initial access, lateral movement, and exfiltration—and techniques, which detail the methods used to achieve these objectives. Each technique may include sub-techniques for further granularity, while specific procedures document real-world implementations. This layered approach enables organizations to understand how adversaries operate and why certain actions are taken, supporting comprehensive threat modeling [40,41]. The framework is structured into several matrices, each representing different operational domains:

1. Enterprise: addresses behaviors against enterprise IT networks and cloud environments.
2. Mobile: focuses on adversary behaviors targeting mobile devices.
3. Industrial Control Systems (ICSs): covers behaviors against industrial control systems.

Each matrix is organized into tactics, which are the adversary’s technical objectives, and techniques, which are the methods used to achieve those objectives. This hierarchical categorization enables cybersecurity professionals to map and predict adversary behavior effectively, thereby enhancing the resilience of digital infrastructures.

#### 2.3.1. Core Components

To effectively understand and address adversarial behaviors, the MITRE ATT&CK framework is built upon several core components that provide a structured approach to categorizing and mitigating cyberthreats. Each component offers unique insights into different aspects of an adversary’s actions, forming a cohesive system for understanding and responding to cyberattacks.

1. Tactics: represent the overarching why behind an adversary’s actions during an attack. They define the adversary’s strategic goals, such as Initial Access, Privilege Escalation, or Defense Evasion. Each tactic serves as a high-level category under which specific techniques are grouped, providing context for an adversary’s behavior across various stages of an attack lifecycle. This categorization allows defenders to align their detection and mitigation efforts with adversarial objectives [42,43].
2. Techniques: describe the how, and the specific methods adversaries use to achieve their objectives within a tactic. For instance, within the Initial Access tactic, techniques like Phishing or Exploitation of Public-Facing Applications explain distinct methods adversaries employ [43,44].
3. Sub-techniques: offer a deeper layer of specificity, breaking down broader techniques into distinct variations. These enable defenders to focus on nuanced adversarial behaviors and tailor their detection strategies accordingly. Sub-techniques illustrate the evolving nature of adversarial actions and enhance the utility of the framework in addressing targeted threats [42].
4. Procedures: describe how techniques or sub-techniques are implemented in real-world scenarios. These include specific tools, commands, or methods adversaries employ during an attack. For instance, a procedure for Command and Control might document the use of HTTPS traffic to obfuscate malicious communication. Understanding procedures aid defenders in recognizing and mitigating adversarial actions as they unfold [43].
5. Mitigation: the strategies address the ’what-to-do’ aspect by proposing defensive measures to counter specific techniques or sub-techniques. For instance, multi-factor authentication is recommended to mitigate the Valid Accounts technique, while network segmentation is effective in addressing Lateral Movement. These recommendations enable organizations to enhance their defensive posture against specific adversarial behaviors, thereby improving their overall security framework. [42,44].

#### 2.3.2. Applications of ATT&CK in Categorizing TTPs

Applications of the MITRE ATT&CK framework in categorizing tactics, techniques, and procedures (TTPs) have transformed how organizations approach cybersecurity. Its applications span adversary emulation, gap analysis, and behavioral analytics, providing a comprehensive approach to understanding and addressing adversarial actions in real-world scenarios.

1. Adversary emulation and threat hunting: the ATT&CK framework serves as a foundational tool for adversary emulation, enabling red teams to simulate real-world attack scenarios based on documented TTPs. Similarly, it supports proactive threat hunting by aligning detection strategies with known adversarial behaviors, enhancing the efficiency of SOCs [43].
2. Mapping and gap analysis: ATT&CK aids in mapping organizational defenses against adversarial TTPs, helping identify coverage gaps, and emphasizes the importance of accurate mapping practices to maximize the framework’s utility [42]. This process involves aligning observed attack data with ATT&CK’s techniques and sub-techniques, ensuring comprehensive coverage of defensive measures.
3. Behavioral analytics development: by integrating ATT&CK into behavioral analytics, organizations can create robust detection mechanisms that identify anomalies aligned with known adversarial actions. This integration improves the speed and accuracy of incident response efforts, reducing potential damage from attacks [44].

### 2.4. NIST Cybersecurity Framework (CSF)

The National Institute of Standards and Technology (NIST) Cybersecurity Framework (CSF) is a cornerstone in addressing the complex and evolving landscape of cyberthreats. Designed as a flexible, voluntary guideline, the NIST CSF aims to enhance the cybersecurity posture of organizations by providing a structured approach to risk management [45].

This framework was intended to address the growing concerns about vulnerabilities in systems essential to national security, economic stability, and public safety [46]. Published in 2014, the framework is built upon industry best practices and incorporates input from a diverse range of stakeholders, making it both robust and adaptable [45].

The NIST CSF comprises five functions: Identify, Protect, Detect, Respond, and Recover. These functions represent the lifecycle of managing cybersecurity risks and provide a high-level taxonomy for organizing cybersecurity activities. Each function is further broken down into categories and subcategories that offer actionable guidance on achieving specific outcomes. For instance, the “Identify” function emphasizes asset management, risk assessment, and governance, forming the foundation for understanding and managing cybersecurity risks. Furthermore, the framework’s tiered implementation model enables organizations to assess their current cybersecurity maturity and set realistic improvement goals based on their resources and risk tolerance [45].

As cyberthreats continue to grow in sophistication and frequency, the NIST CSF serves as a vital tool for fostering resilience and adaptability. By providing a structured yet flexible approach to cybersecurity, the framework empowers organizations to address current risks while preparing for future challenges.

### 2.5. Large Language Models for Cybersecurity

Cybersecurity is a dynamic field requiring timely detection and response to threats. With the increasing sophistication of cyberattacks, traditional tools often fall short in addressing complex, evolving challenges. Large language models (LLMs) have emerged as transformative tools, enabling automation and augmentation of tasks such as malware detection, phishing prevention, and threat intelligence analysis [47].

This study investigates and compares the contributions of prominent LLMs, including general-purpose models like GPT-4, T5, and LLaMA, and domain-specific models such as CyberBERT, and Pixtral 1.2B. Special attention is given to their applicability in cybersecurity tasks, such as log analysis, incident response, and contextualizing threat intelligence.

1. GPT-4: Large language models (LLMs) like GPT-4 have shown immense potential in advancing cybersecurity workflows, particularly in tasks such as summarizing threat intelligence reports and analyzing phishing emails. These models leverage their extensive knowledge base and human-like text generation to effectively process and interpret complex textual data. However, when applied to more technical challenges, such as binary reverse engineering or the analysis of highly detailed logs, GPT-4 often encounters limitations. Studies by Wu, Fangzhou et al. [48], and Pordanesh, Saman, et al. [49] reveal that while GPT-4 performs well in general software security tasks, its ability to handle nuanced and domain-specific problems diminishes without fine-tuning. These findings underscore the need for targeted training and domain adaptation to fully harness GPT-4’s capabilities for specialized cybersecurity applications, bridging the gap between general language understanding and technical precision.
2. T5 (Text-to-Text Transfer Transformer): The T5 model’s text-to-text architecture has demonstrated effectiveness in translating log data into actionable insights, with successful applications in parsing security documentation, generating attack scenario summaries, and conducting vulnerability assessments. However, deploying T5 in real-time cybersecurity scenarios necessitates optimization for speed [50]. Additionally, research on full-stack optimization of transformer inference provides insights into improving efficiency for real-time deployment [51]. These advancements are crucial for implementing T5 in real-time cybersecurity contexts, where rapid response is essential.
3. LLaMA: Meta’s LLaMA models have demonstrated strong performance in reasoning and analytical tasks. For instance, the LLaMA 3.3-70B model exhibits significant improvements in handling complex analytical tasks, enhancing its reliability for problem-solving across various domains [52].
  However, applying LLaMA in specialized domains like cybersecurity presents challenges. A comprehensive review of generative AI and large language models for cybersecurity highlights that while LLaMA models offer potential benefits, their deployment requires significant customization to effectively address domain-specific tasks [53].
4. CyberBERT: CyberBERT, a model fine-tuned from BERT for cybersecurity applications, has demonstrated proficiency in tasks such as malware classification, log analysis, and phishing pattern identification. For instance, the SecureBERT model, a domain-specific adaptation of BERT, has shown significant improvements in predicting masked words within cybersecurity texts, outperforming models like RoBERTa and SciBERT [54].
  However, these models primarily excel in discriminative tasks, such as classification and sequence labeling, due to their training objectives. Their generative capabilities are limited, which poses challenges in applications requiring text generation, such as automated report writing or threat scenario simulation. This limitation is inherent in BERT-based architectures, which are designed for understanding and classification rather than generation [55].
5. Pixtral 12B: Mistral AI’s Pixtral 12B is an open-source multimodal model with 12 billion parameters, excelling in both image and text data processing. Its architecture combines the Mistral Nemo text model with a custom vision encoder, enabling it to handle a wide array of tasks, including instruction following and text-only benchmarks [56].
  While Pixtral 12B’s versatility positions it as a valuable tool across various domains, specific applications in cybersecurity, such as intrusion detection and phishing email classification, would require fine-tuning the model with domain-specific data to enhance its performance in these specialized tasks. Techniques like Low-Rank Adaptation (LoRA) [57] can be employed for efficient fine-tuning, allowing the model to adapt to custom datasets without extensive computational resources [58].
  Additionally, Pixtral 12B’s compatibility with cloud services and NVIDIA GPUs facilitates faster deployments, making it suitable for resource-constrained environments. Its open-source nature and support for variable image sizes further contribute to its adaptability in diverse operational settings [56].

In this study, we employed GPT-4o and Pixtral 12B as the foundational models for our Security Event Response Copilot (SERC) system due to their complementary capabilities and potential to address critical challenges in cybersecurity operations. GPT-4o stands out for its exceptional language understanding, contextual reasoning, and ability to process complex textual data, making it particularly well suited for tasks such as interpreting threat intelligence reports, providing actionable insights, and guiding incident response workflows.

On the other hand, Pixtral 12B was selected for its multimodal capabilities and flexibility in processing both text and structured data. Its architecture excels in instruction-following and multimodal data interpretation, enabling it to process diverse inputs such as log data, system alerts, and visual representations of threats. This versatility is critical in dynamic SOC environments where security analysts must correlate information from various sources to develop a comprehensive understanding of incidents.

### 2.6. Architecture of RAG Frameworks

Retrieval-Augmented Generation (RAG) is a framework that enhances generative language models by integrating external retrieval mechanisms, thereby improving the accuracy and relevance of AI-generated outputs. This approach addresses limitations such as hallucinations and outdated knowledge inherent in standalone generative models [59].

At its core, RAG comprises two main components: a retriever and a generator. The retriever identifies pertinent documents or information from an external knowledge base, while the generator synthesizes this information into coherent responses. This dynamic interaction allows RAG systems to access up-to-date and domain-specific knowledge, extending their applicability to specialized fields where information is continually evolving [59]. This dynamic interplay between retrieval and generation not only improves the factual accuracy of generated outputs but also extends the applicability of such systems to specialized domains where knowledge is continuously evolving [60].

**Figure 2:**
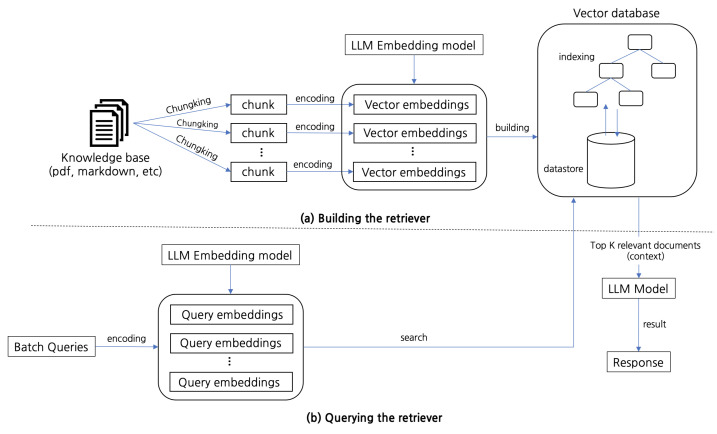
Overview of Retrieval-Augmented Generation.

The architectural pipeline of RAG is visually represented in Figure 2, which delineates the retriever and generator components. The process begins with the retriever, as outlined in the top section of the figure. A knowledge base comprising various documents, such as PDFs or markdown files, is chunked into smaller segments to facilitate retrieval. Each chunk is then encoded into vector embeddings using a pre-trained LLM embedding model. These embeddings are indexed and stored in a vector database, allowing for efficient similarity-based searches. During querying, input queries are encoded into embeddings, which are then used to search the database for the top K most relevant documents. This retrieval mechanism ensures that only the most pertinent information is passed to the generator.

The generator is represented in the lower part of the figure, where the retrieved documents serve as context for the LLM Model. This model synthesizes the retrieved information and the query context to produce a coherent and contextually accurate response. The output, labeled as “Response”, represents the final step in the RAG pipeline. By combining retrieval with generation, this architecture leverages external knowledge bases to overcome the static limitations of standalone generative models, resulting in outputs that are both context-aware and factually grounded.

### 2.7. Role of Large Language Model Copilots in Enhancing Efficiency, Personalization, and Cybersecurity

Large language models (LLMs), with their advanced natural language processing (NLP) capabilities, have emerged as transformative technologies, automating complex workflows and providing contextualized assistance. Their integration as copilots has significantly enhanced productivity across domains such as academic research, software development, and cybersecurity, demonstrating their potential to augment human decision-making.

Recent studies have explored the diverse applications of LLM copilots in various fields. Lin et al. [61] introduced an LLM-based system for personalized academic assistance, utilizing thought-retrieval mechanisms and user profiling to offer tailored support to researchers, thereby saving considerable time in academic workflows. Similarly, Li et al. [62] presented a framework leveraging LLM agents for autonomous machine learning research, facilitating tasks such as idea generation, experiment design, and execution, and showcasing their ability to automate complex scientific explorations. Furthermore, Wen et al. [63] developed OverleafCopilot, an extension of the Overleaf academic writing platform, which streamlines writing, editing, and formatting processes through LLM integration.

In cybersecurity, tools like Fortinet’s Advisor exemplify the application of generative AI to streamline Security Operations Center (SOC) processes, providing real-time assistance with triage, investigation, and incident response [64]. Complementing commercial tools, academic research underscores the utility of LLMs in automating repetitive tasks, generating enriched alerts, and enabling natural language querying [13].

By integrating LLM capabilities into Wazuh’s extensible framework, we developed a copilot solution that mirrors the functionalities of commercial tools while promoting community-driven innovation and cost-effectiveness [65]. This study proposes a framework for Wazuh Copilot, an open-source, LLM-powered assistant designed to enhance Wazuh deployments, leveraging Wazuh’s SIEM and XDR features, combined with generative AI functionalities.

### 2.8. Challenges When Using LLM in SOC Space

The escalating complexity and frequency of cyberthreats have placed considerable demands on Security Operations Centers (SOCs) to identify, analyze, and respond to incidents rapidly and accurately. Traditional approaches often rely heavily on manual analysis, which is time-consuming and can lead to alert fatigue among security analysts. Recent advances in artificial intelligence, particularly with large language models (LLMs), have introduced new possibilities for automating and enhancing SOC operations. LLMs, known for their proficiency in processing and generating human language, offer promising applications in cybersecurity where rapid understanding of diverse data sources—such as security logs, threat reports, and alerts—is essential for effective incident response as suggested by Yao et al. [66]. The integration of LLMs within SOCs can streamline threat intelligence analysis, improve alert prioritization, and reduce the workload on security analysts, allowing them to focus on high-priority incidents.

However, traditional methods for adapting LLMs to specific domains, such as fine-tuning, come with limitations. Fine-tuning an LLM involves retraining the model on domain-specific data, which enables it to specialize in understanding cybersecurity language. While this approach enhances relevance, it is computationally intensive and requires frequent retraining to stay effective as new threats emerge [67,68]. Additionally, fine-tuned models may still struggle to incorporate the most recent threat intelligence, as their knowledge base is fixed post-training [69].

To address these limitations, researchers have turned to Retrieval-Augmented Generation (RAG), an approach that enhances LLMs by dynamically retrieving relevant information from external knowledge sources during inference. RAG combines an LLM with a retrieval component that pulls information from an up-to-date database or knowledge base, allowing the model to produce responses based on the latest available information. This capability makes RAG particularly well suited for SOC environments, where cybersecurity threats evolve rapidly, and the need for current intelligence is critical [70,71].

In contrast to fine-tuning, RAG enables a more flexible and scalable solution for SOCs. RAG allows organizations to maintain an LLM’s core language capabilities while continuously feeding it real-time threat intelligence, without the overhead of retraining the model itself [71]. This integration of external data sources during inference supports SOC analysts by providing timely, context-rich responses, which enhances situational awareness and facilitates more accurate and responsive decision-making. By focusing on RAG, this paper seeks to address key challenges in incident response, such as alert fatigue and data relevance, by harnessing real-time data retrieval to produce actionable intelligence.

Building on the foundational studies by Yao et al. [66] and Xu et al. [72], the study explores how RAG can be leveraged within SOC workflows to overcome the limitations of fine-tuned LLMs and achieve a higher degree of adaptability and efficiency. The implementation of RAG in SOCs aims to establish a robust framework that not only improves detection accuracy but also enables swift, data-driven responses that are essential for mitigating today’s sophisticated cyberthreats.

Tseng et al. [73] explore the integration of LLMs into SOC workflows, demonstrating how these models can be leveraged to enhance response accuracy and efficiency. By automating critical aspects of analysis and providing context-aware recommendations, LLMs reduce the cognitive load on analysts and accelerate decision-making during security incidents [53,74].

### 2.9. Atomic Red Team: A Modular Framework for Adversary Simulation and Detection Validation

In the evolving landscape of cybersecurity, adversary emulation has become a cornerstone for assessing the robustness of detection and response capabilities. The Atomic Red Team project, spearheaded by Red Canary, has emerged as a pivotal open-source framework designed to empower security teams with a library of lightweight, modular tests that emulate real-world adversary tactics and techniques [75].

Atomic Red Team fills a critical gap in cybersecurity operations by providing security teams with a practical, accessible, and standardized approach to adversary emulation. Unlike traditional penetration testing, which often lacks alignment with tactical frameworks, Atomic Red Team tests are structured to replicate specific techniques such as privilege escalation, credential dumping, and lateral movement. This approach ensures that detection and response mechanisms are thoroughly tested in a controlled yet realistic manner [76].

Security professionals can implement and execute these tests with minimal configuration, enabling continuous validation of security controls without significant operational disruption. Moreover, the framework’s open-source nature fosters collaboration and innovation, allowing contributors to enhance their library and adapt tests to evolving threat landscapes [77].

## 3. Methodology

### 3.1. Proposed RAG Components

The proposed RAG system integrates advanced components to facilitate efficient data retrieval and processing. It embeds data using the BAAI/bge-large-en-v1.5 model, a sophisticated embedding model optimized for creating high-dimensional vector representations of textual data. For vector storage and similarity search, it employs Qdrant, a robust vector database designed for managing embeddings, leveraging cosine similarity as the metric for identifying semantically relevant matches. The preprocessing pipeline begins with raw event logs in formats like JSON, which are parsed and encoded using a prompting LLM extractor powered by the Pixtral model. This model dynamically extracts and structures critical information based on predefined beneficial criteria, ensuring the extracted data align with retrieval objectives. The parsed information is then encoded into embeddings for efficient storage and retrieval within the vector database, enabling seamless integration of retrieval-augmented workflows in downstream applications.

#### 3.1.1. Qdrant Vector Database

Among the many vector database solutions, Qdrant stands out as an open-source, scalable, and production-ready platform tailored for vector similarity search and hybrid retrieval tasks [78]. Unlike conventional search engines, Qdrant facilitates dense vector searches, providing a robust infrastructure for applications in semantic search, content recommendation, and RAG systems. Its integration capabilities with popular machine learning frameworks make it a preferred choice for both academic and industrial applications.

Furthermore, Qdrant’s support for Retrieval-Augmented Generation (RAG) has proven instrumental in augmenting large language models (LLMs) with domain-specific knowledge through vectorized document retrieval [79]. This integration empowers LLMs to generate context-aware and domain-specific responses.

#### 3.1.2. Embedding Model: BAAI/bge-large-en-v1.5

The open-source embedding model BAAI/bge-large-en-v1.5 from Hugging Face was utilized to transform text data into vector representations. This model is optimized specifically for English text, making it well suited for RAG and information retrieval applications within the cybersecurity domain. The BAAI/bge-large-en-v1.5 model offers notable advantages [70,80]:

1. Efficiency and scale: the model demonstrates high efficiency in processing large volumes of text data while maintaining high accuracy in similarity detection. It uses a technique known as Matryoshka Representation Learning [81], which enables the model to generate embeddings in multiple dimensions (e.g., 1024, 768, 512, down to 64 dimensions) without significant loss of accuracy.
2. Performance comparison: compared to similar models such as OpenAI’s Ada [82] and traditional BERT-based models [83], BAAI/bge-large-en-v1.5 is optimized for faster responses and adaptability with large datasets. Its open-source nature also makes it more cost-effective than proprietary models, which is an important consideration for scalable cybersecurity applications.

#### 3.1.3. Similarity Metric: Cosine Distance

Surrounded by various similarity measures, cosine similarity and its counterpart, cosine distance, have emerged as popular metrics due to their focus on the angular relationship between vectors, independent of magnitude. This attribute has made cosine-based measures highly effective in applications such as document retrieval, text classification, and embedding evaluations [84,85].

The formula for cosine similarity is mathematically expressed as:

$$s=\cos\theta =\frac{A\cdot B}{∥A∥\times ∥B∥}=\frac{\sum_{i=1}^{n}A_{i}B_{i}}{\sqrt{\sum_{i=1}^{n}A_{i}^{2}}\times \sqrt{\sum_{i=1}^{n}B_{i}^{2}}}$$

where

- A and B are the vectors being compared;
- A·B is their dot product;
- ∥A∥ and ∥B∥ are the magnitudes of the vectors.

Cosine similarity is normalized, meaning it is independent of vector magnitude, making it well-suited for applications where the scale of the data varies significantly. The similarity score *s* ranges from −1 to 1, where 1 indicates perfect similarity, 0 denotes orthogonality (no similarity), and −1 represents completely opposite vectors [86].

### 3.2. RAG Workflow

#### 3.2.1. Augmentation Process

**Figure 3:**
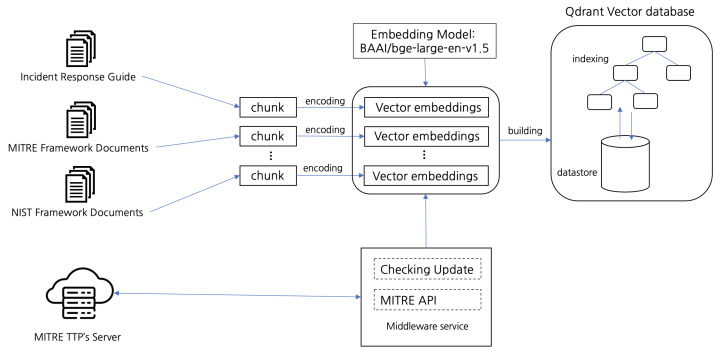
Augmentation process illustration.

To facilitate the effective retrieval of documents relevant to cybersecurity incidents, as illustrated in Figure 3, three distinct collections, as detailed in Table 2, were created within the vector database. Each collection was specifically designed to serve a unique function within the RAG model, ensuring that query results were both contextually relevant and aligned with industry-standard frameworks.

Drawing on these authoritative sources, the integrated corpus combines an Incident Response Guide and Plan, the updated CSF 2.0, and the MITRE ATT&CK framework. Together, these documents provide procedural guidance for addressing breaches, strategic oversight through the structured approach in CSF, and detailed adversarial insights from MITRE ATT&CK.

By maintaining each reference in a separate, complementary collection within the vector database, the RAG model can dynamically surface context-specific guidance, whether for immediate incident containment, alignment with recognized cybersecurity standards, or the identification of adversary tactics and techniques. Consequently, each query is grounded in industry-standard guidelines and real-world threat scenarios, thereby enhancing both the relevance and reliability of the model’s output.

1. General knowledge: this collection provides foundational knowledge for computer security incident handling, derived from sources such as security operations and automation response (SOAR) playbooks and general cybersecurity incident handling guides. It serves as a primary resource for addressing general security incident management needs.
2. NIST knowledge: specifically structured to align with the NIST Cybersecurity Framework, it includes guidelines, policies, and standards that ensure relevance to regulatory frameworks and assist in the compliance verification process.
3. MITRE knowledge: designed around the MITRE ATT&CK framework, this collection supports the retrieval of documents directly relevant to threat mitigation and response strategies. By mapping queries to specific MITRE tactics and techniques, this collection enhances the precision of actionable insights for mitigating cyberthreats. To ensure that the collection remains aligned with the state-of-the-art MITRE tactics and techniques, a dedicated service periodically checks the MITRE servers for updates and synchronizes the local collection in the vector database, enabling real-time adaptability to emerging threats and maintaining the relevance of the system.

#### 3.2.2. Chunking Techniques

Based on observations, two types of documents were identified for storage in the database as primary references for the RAG system. For instance, guideline books containing multiple subtopics with cross-contextual information were processed by segmenting them into smaller chunks using the Natural Language Toolkit (NLTK) splitter [94], ensuring each chunk was contextually coherent.

In contrast, a single document encompassing a specific use case, attack context, mitigation strategies, and response procedures could be stored as a unified index. This approach ensured the preservation of contextual integrity and provided a comprehensive guideline for effective mitigation and incident response. This approach ensured that the system could retrieve documents accurately and consistently with the correct contextual alignment in future queries.

#### 3.2.3. Large Language Model Configuration

In this study, we configured the AI model parameters, as shown in Table 3, to optimize the model’s performance and ensure consistent recommendation accuracy. This setup was determined through iterative experimentation, guided by our objectives of maintaining model stability and achieving uniform behavior across diverse inputs.

### 3.3. Wazuh Event Security Data Extraction and Refinement

Upon detecting a vulnerability attack, Wazuh captures critical details using the rules-based matching method [95] and produces a security event log in JSON format [96], a common data exchange format that organizes data into hierarchical key-value pairs, facilitating efficient storage and retrieval. While JSON’s structured and metadata-rich format is well suited for computational tasks, it requires transformation into a more contextually enriched format to be effectively processed by large language models (LLMs). Consequently, aligning the structured representation of JSON into a format that preserves its semantic and contextual integrity is a critical preprocessing step in Retrieval-Augmented Generation (RAG) pipelines [97]. This transformation ensures that the data are optimized for downstream retrieval and reasoning tasks.

Wazuh generates two types of security event logs, those containing an MITRE ID and those without [98]. The refined process ensures that key metadata such as associated MITRE tactics, alert severity, compliance concerns, and detailed log descriptions are extracted and presented in a structured format. Such an approach enhances the accuracy and relevance of retrieval in RAG systems, enabling effective cybersecurity responses. By enriching the logs with actionable insights, the system ensures alignment with cybersecurity frameworks and provides critical information for threat mitigation.

This approach ensures that all logs, regardless of their initial structure, are transformed into coherent and actionable entries that align with cybersecurity frameworks like MITRE ATT&CK. The refined outputs are subsequently used to construct prompts for large language models (LLMs), enabling accurate and context-aware retrieval of information for threat detection and mitigation.

**Figure 4:**
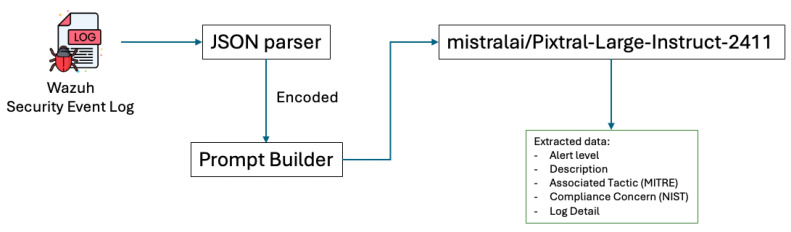
Event security data extraction pipeline.

The diagram illustrated in Figure 4 shows a structured pipeline for transforming JSON-formatted input logs into determined plain text. In order to refine the data, the process involves several steps. First, the raw JSON event log is parsed into an encoded JSON format, preparing the data for subsequent handling. Next, the encoded JSON is embedded into a user prompt, as illustrated in Table 4. This step employs a one-shot output approach to ensure consistency in the format of the generated responses.

This refined, text-based structure facilitates more efficient tokenization and compatibility with vector-based similarity searches in vector databases, enhancing the retrieval process within RAG systems. By translating structured JSON logs into a format optimized for vector-based systems, this method supports more accurate and contextually relevant document retrieval, enabling SOC analysts to access real-time, actionable intelligence for enhanced threat detection and incident response.

### 3.4. Retrieval Methodology for Security Event Log Analysis and Response

**Figure 5:**
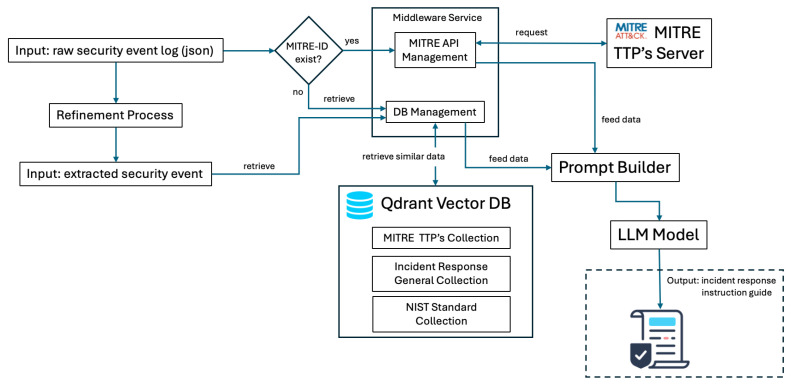
Security Event Response Copilot (SERC) Workflow.

The copilot system was designed to respond by analyzing the extracted data for the presence of a given MITRE ID and subsequently retrieving relevant contextual information directly from a server or database. This retrieval process involved querying three distinct sources: the MITRE ATT&CK framework, general incident response guidelines, and the NIST Cybersecurity Framework (CSF) collection, as detailed in Table 2. These three data sources played a critical role in ensuring the relevance and accuracy of the system’s output. The retrieved information was then consolidated and served as input for the prompt builder, which integrated the contextual data to construct a coherent and contextually informed prompt for the AI model. Leveraging this enriched context, the AI model generated a tailored instruction guide that was delivered to the user. This structured workflow shown in Figure 5 ensured that the copilot system provided precise, actionable, and context-aware guidance, enhancing its utility in real-time incident response and decision-making scenarios.

The SERC was further equipped with a chat response feature designed to facilitate follow-up interactions for specific cases related to the initial response. This feature enabled a dynamic and iterative communication process, allowing users to query the system for additional details, clarifications, or guidance tailored to the nuances of a given incident. By integrating conversational capabilities, the SERC enhanced its utility as a responsive and adaptive tool, ensuring that users received comprehensive support throughout the incident management lifecycle. This functionality is particularly valuable in addressing complex scenarios where continuous interaction is required to refine and implement effective cybersecurity measures.

As outlined in Table 5, the response format is utilized as an input prompt for the model, integrating comprehensive knowledge from general incident response guidelines, the NIST Cybersecurity Framework (CSF), and the MITRE framework. This approach facilitates the generation of contextually relevant mitigation follow-up actions tailored to specific security incidents.

## 4. Experiment Results

### 4.1. Copilot Integration for Security Event Analysis in Wazuh

**Figure 6:**
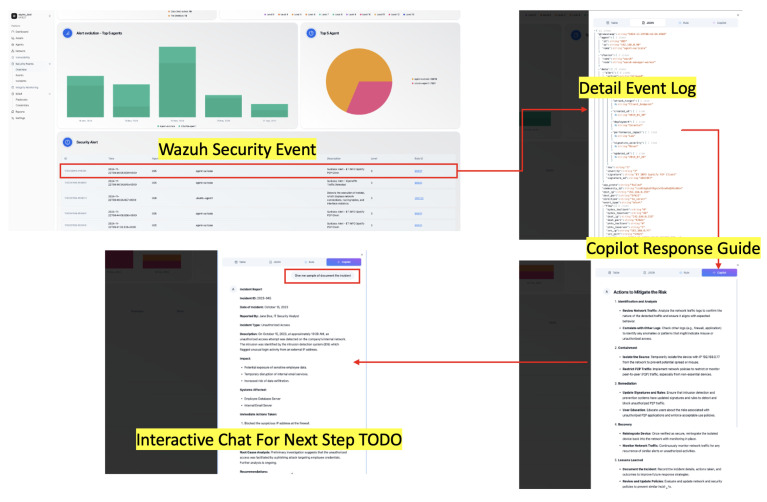
Copilot embedded into Wazuh with custom interface.

The custom dashboard presented in Figure 6 illustrates the implementation of a streamlined workflow for managing security events within the Wazuh platform. The image depicts several key features that enhance user interaction and event analysis. The primary section of the dashboard shows a detailed list of security events detected by Wazuh. A new tab labeled Copilot is prominently featured in the event details section. This tab serves as an interface for leveraging the advanced analytical capabilities of the SERC (Security Event Response Copilot) system.

The results from the SERC system are rendered in real time within the Copilot tab. This enables users to view critical findings, such as risk assessments, recommended actions, or contextual insights, directly within the Wazuh interface without switching applications. A dynamic chat section is integrated into the Copilot, allowing users to interact with the system by providing additional instructions or querying specific aspects of the processed data. This feature enhances the decision-making process by enabling a tailored response to the presented insights.

### 4.2. Simulation Scenario

**Figure 7:**
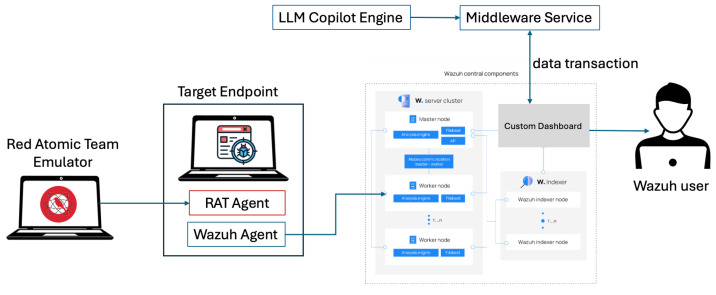
High-level simulation workflow.

This study conducted an end-to-end simulation of attack and defense mechanisms to evaluate the integration of automated detection and mitigation procedures, as illustrated in Figure 7. The framework utilized the Atomic Red Team tool to simulate predefined adversarial scenarios, meticulously designed with anticipated outcomes. These scenarios were executed on a monitored target endpoint equipped with Wazuh agents, enabling the generation of relevant security events for analysis.

Detected security events were processed to initiate user interaction through a copilot interface, providing tailored mitigation guidelines aligned with the attack types defined in the MITRE ATT&CK framework.

**Figure 8:**
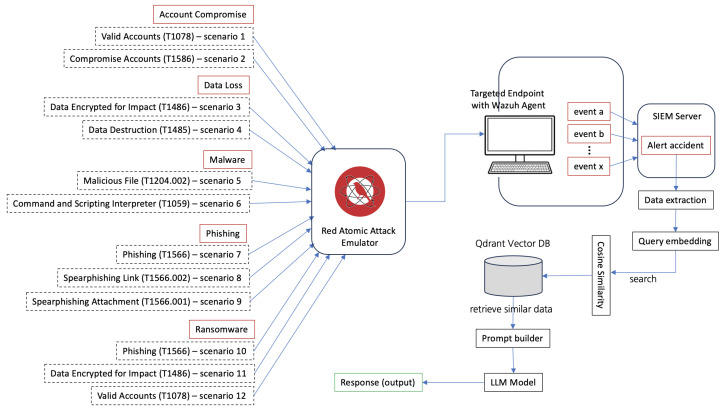
Attack simulation scenario.

This workflow, as illustrated in Figure 8, allows users to respond effectively by adhering to context-specific instructions. The selected attack scenarios provide precise measurements of inputs (attack actions) and outputs (detection and response), thereby enabling a comprehensive assessment of the system’s performance under realistic conditions.

The experiment employed the SOCFortress Incident Response Plan [99], a repository renowned for its alignment with real-world threat scenarios, as a robust benchmark. Adversarial actions were simulated using the Red Atomic Attack Emulator in conjunction with the MITRE ATT&CK framework. Security events generated during the attacks were collected and cataloged by the Wazuh SIEM system. This setup facilitated the evaluation of two prominent language models OpenAI and Pixtral for their capacity to process and respond effectively to simulated cyberattacks.

#### Setting the Stage: Building the Simulation

Our experiment focused on six distinct attack types, each carefully designed to mimic realistic scenarios. The categories Account Compromise, Data Loss, Malware, Phishing, and Ransomware were selected based on our ground truth [99], as they represent some of the most critical threats in modern cybersecurity. Each attack was systematically mapped to specific MITRE ATT&CK techniques, including “Valid Accounts” (T1078), “Data Encrypted for Impact” (T1486), and “Malicious File” (T1204.002). This approach ensured that the simulation adhered to standardized methodologies while reliably generating the expected security events, establishing a solid foundation for evaluating detection and response mechanisms.

To achieve this, the simulation framework was designed to reliably trigger corresponding alerts in the Wazuh SIEM system, meeting the necessary conditions for detection and response. For example, the “Valid Accounts” technique led to successful credential misuse, generating authentication-related logs, while “Data Encrypted for Impact” created file system events indicative of ransomware activity. Similarly, “Malicious File” events were crafted to simulate malware execution, producing alerts that highlighted anomalous process behaviors and file manipulations.

This alignment between simulated attack actions and expected security events was critical to validating the accuracy and completeness of the detection system. By ensuring that every attack type generated its intended security events, the experiment provided a robust foundation for evaluating how well-automated detection and response mechanisms could process and address these threats in real-time.

We turned to the Red Atomic Attack Emulator, a tool capable of generating precise and controlled attack scenarios [100]. As the attacks unfolded, the Wazuh agent, installed on a targeted endpoint, captured the resulting security events. These events, flowing into the SIEM server, formed the heartbeat of our experiment, a stream of alerts and logs ready for analysis.

### 4.3. Performance Evaluation

To evaluate the effectiveness of our proposed simulation framework, we undertook a meticulous comparison of the recommendations generated by the copilot system against a carefully curated ground truth [99]. The evaluation journey unfolded through the use of several well-established metrics, each chosen for its unique ability to assess different dimensions of text quality and alignment.

Our first step was to employ cosine similarity, a measure that allowed us to quantify the semantic alignment between the copilot’s recommendations and the ground truth. By transforming the texts into vector representations and calculating their similarity, this method revealed how closely the generated outputs adhered to the intended meaning.

Building upon this, we turned to BERTScore [101], which offered a deeper insight into contextual accuracy. Leveraging the power of transformer-based embeddings, this method allowed us to capture subtle nuances in meaning and determine whether the copilot’s responses truly reflected the intent of the ground truth. The rich contextual evaluation provided by BERTScore complemented the semantic perspective of cosine similarity.

This multi-faceted evaluation approach enabled us to delve into the performance of the copilot system from multiple perspectives: semantic, contextual, structural, and recall-based. The combined insights demonstrated the system’s capability to align with the ground truth and highlighted areas for potential refinement, further reinforcing the robustness of the proposed simulation framework in replicating real-world cybersecurity response scenarios.

**Figure 9:**
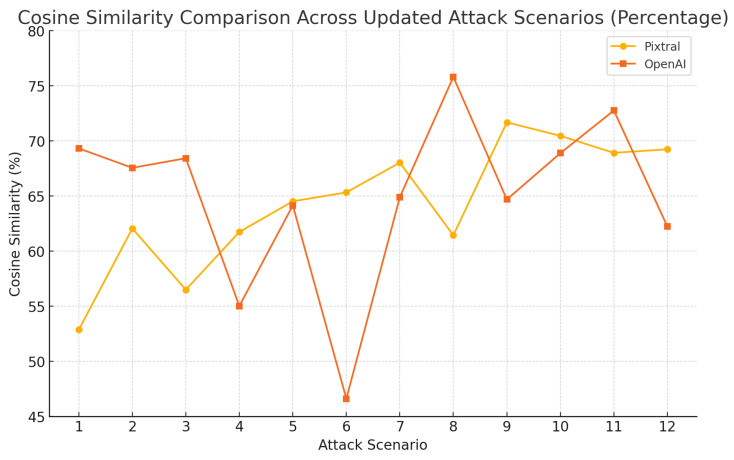
Model performance from various attacks using cosine similarity.

The results shown in Figure 8 illustrate the attack flow and the corresponding scenarios, while the cosine similarity scores for Pixtral and OpenAI across these 12 attack scenarios are presented in Figure 9. OpenAI generally maintained a higher or comparable level of semantic alignment with the ground truth, peaking at certain scenarios (e.g., scenario 8 with 75.81%), while exhibiting notable drops in others (e.g., scenario 6 with 46.61%). Pixtral, while more consistent across scenarios, tended to trail OpenAI but showed competitive alignment in specific scenarios, such as 9 and 10.

OpenAI consistently achieved higher percentages in most scenarios, peaking at 75.81% in scenario 8, while Pixtral exhibited steadier performance, reaching up to 71.69% in scenario 9

**Figure 10:**
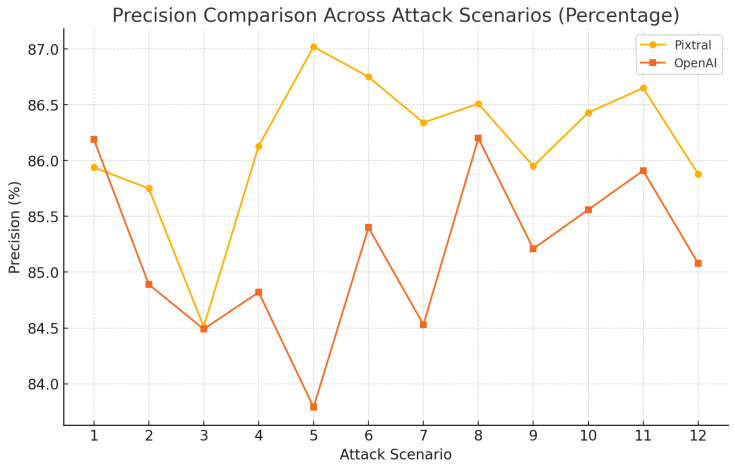
Model performance from various attacks using BERTScore: precision.

As shown in Figure 10, Pixtral consistently maintained higher precision percentages, ranging between 84.5% and 87.0%, with minimal fluctuations. OpenAI demonstrated more variability, with percentages dipping below Pixtral in several scenarios but occasionally achieving competitive values.

**Figure 11:**
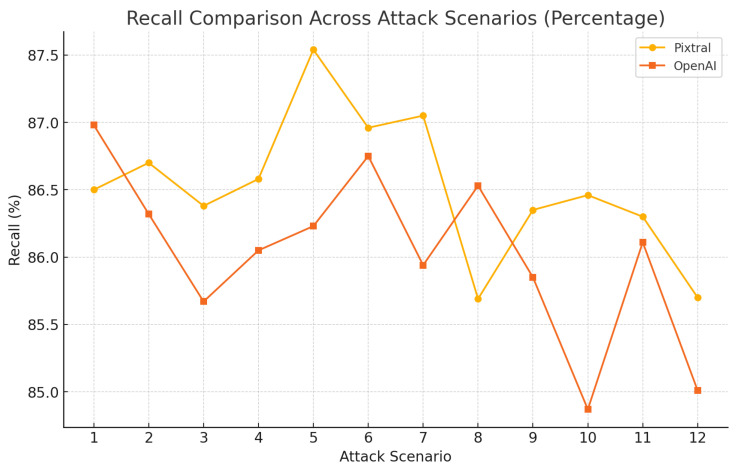
Model performance from various attacks using BERTScore: recall.

Pixtral shows in Figure 11 slightly higher recall percentages in most scenarios, ranging from 85.6% to 87.5%, reflecting its reliability in capturing relevant information. OpenAI, while competitive, displays greater variability, with recall percentages fluctuating between 84.8% and 86.9%.

**Figure 12:**
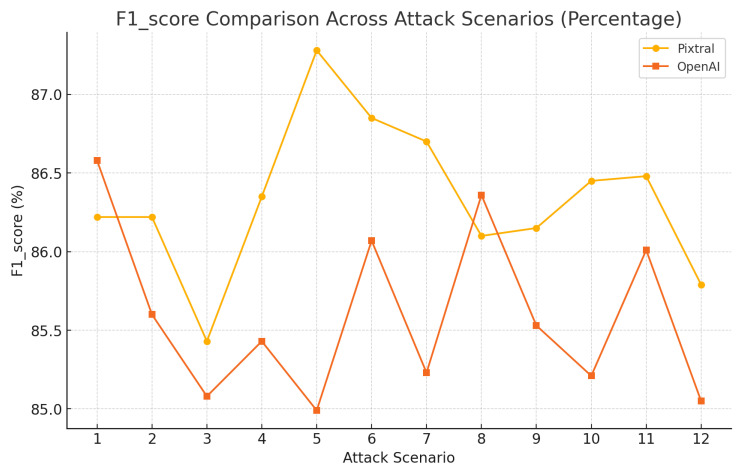
Model performance from various attacks using BERTScore: F1.

Pixtral’s F1-score percentages, as shown in Figure 12, consistently ranged between 85.4% and 87.2%, showcasing balanced performance across scenarios, while OpenAI, capable of achieving comparable F1 scores in certain scenarios, exhibited more pronounced dips, with percentages ranging between 84.9% and 86.5%.

## 5. Discussion and Future Work

This study revealed distinct patterns in the performance of Pixtral and OpenAI when tasked with aligning generated responses to the ground truth across a variety of attack scenarios. Pixtral consistently demonstrated reliability, maintaining stable precision, recall, and F1 scores throughout the evaluation. This consistency reflects its ability to generate responses that are both relevant and comprehensive, making it a dependable choice in diverse contexts. However, while Pixtral’s steadiness is an asset, its performance plateaued in certain scenarios, limiting its potential to achieve the highest possible alignment with the ground truth.

In contrast, OpenAI exhibited a broader range of performance. It achieved competitive scores in specific scenarios, showcasing its adaptability and potential for strong contextual understanding. However, its performance was more variable, with noticeable dips in recall and F1 scores in certain attack scenarios. This inconsistency suggests challenges in balancing precision and recall, which are critical for generating comprehensive and relevant responses in cybersecurity contexts.

The comparison underscores the complementary strengths of the two models. Pixtral’s steadiness ensures dependable performance, while OpenAI’s adaptability highlights its potential for excelling in nuanced scenarios. Yet, both models demonstrate room for improvement, particularly in scenarios where alignment with the ground truth proved more challenging.

Building on these findings, future research will focus on fine-tuning Pixtral and OpenAI models using domain-specific datasets to improve contextual understanding and alignment with the ground truth across diverse attack scenarios. Priority will be given to scenario-specific training for nuanced threats such as phishing and ransomware to enhance adaptability. Robustness and continuous improvement will be achieved through adversarial training and active learning, incorporating feedback from domain experts. Additionally, the simulation framework will be extended to integrate with Security Orchestration, Automation, and Response (SOAR) systems, enabling end-to-end automation of the incident response lifecycle. This integration aims to enhance the practical utility of AI-driven copilots in Security Operations Center (SOC) environments, streamlining and improving incident management processes.

## Figures and Tables

**Table 1:** Comparative analysis of SIEM solutions.

Feature	Wazuh	IBM QRadar	Splunk-SIEM	Security Onion	GrayLog
Cost [26]	Free	High	High	Free	Free core
Log management [27]	Comprehensive	Strong	Very strong	Good	Good
FIM [28]	Yes	Limited	Limited	Limited	Limited
Integration [29]	High	High	Very High	Moderate	Moderate
Deployment [30]	Moderate	Complex	Moderate	Moderate	Easy
Scalability [31]	High	High	High	Moderate	Moderate
Community [32]	Strong	Moderate	Moderate	Strong	Moderate

**Table 2:** Categories and related content.

Category	Content
General knowledge	Incident Response Guide [87,88,89,90] IRP [91]
NIST knowledge	CSF 2.0 [92]
MITRE knowledge	MITRE ATT&CK [93]

**Table 3:** Configuration parameters for model generation.

Parameter	Value
Temperature	Set to 0.1, ensuring high determinism by minimizing randomness in the model’s predictions, favoring the most probable outputs.
Top-k	Configured as 50, restricting token sampling to the top 50 most likely candidates, reducing the likelihood of low-probability tokens.
Top-p (Nucleus Sampling)	Set to 0.9, allowing dynamic token selection by considering tokens with a cumulative probability of 90%, ensuring a balance between determinism and contextual diversity.
Max Tokens	Defined as 4096, specifying the upper limit for the total number of tokens in the generated output, suitable for applications requiring concise yet comprehensive responses.

**Table 4:** One-shot prompt format.

Prompt Format
Extract the primary issue or problem from the following Wazuh JSON log.
Focus on details like the alert description, severity level, associated tactics, compliance tags, and any specific event data that
clarifies the problem:
{input_event_json}
Present the extracted issue in a concise format, describing the main problem indicated in the log.
Expected Output Example:
”’
Given the JSON log provided, here’s how the response might look:
Extracted Problem:
Description: Wazuh agent ‘suricata-nids’ has stopped, indicating a potential disruption in NIDS monitoring.
Alert Level: 3 (Medium severity)
Associated Tactic: Defense Evasion (MITRE ID: T1562.001—Disable or Modify Tools)
Compliance Concerns: PCI DSS (10.6.1, 10.2.6), HIPAA (164.312.b), TSC (CC7.2, CC7.3, CC6.8), NIST 800-53 (AU.6, AU.14, AU.5), GDPR (IV_35.7.d)
Log Details: Full log message reads “Agent stopped: ’suricata-nids->any’,” suggesting possible interruption in security monitoring.
”’
Expected output above focuses on the core issue, making it easily readable and actionable for SOC and RAG systems.

**Table 5:** Security Event response prompt format.

Response Prompt Format
This is the context information for general knowledge purposes:
{context_general}
This is the context information knowledge of planning for generating the incident response playbook based on The NIST Cybersecurity Framework (CSF) 2.0:
{context_nist}
This is the context information knowledge from MITRE ATT&CK for security incident response mitigation purposes:
{context_mitre}
Based on the above context information, hope you can use and elaborate on the knowledge you have to analyze this incident and tell me what action to take:
json
Based on that incident, what should be done to mitigate the risk? Make sure to use knowledge of the NIST CSF 2.0 and the MITRE ATT&CK
framework to identify the tactics and techniques associated with the incident.
Do not mention the source of JSON or text input, just tell what action to take with Markdown format.

## Data Availability

Data is contained within the article.
